# Time-varying reproduction number estimation: fusing compartmental models with generalized additive models

**DOI:** 10.1098/rsif.2024.0518

**Published:** 2025-01-29

**Authors:** Xiaoxi Pang, Yang Han, Elise Tressier, Nurin Abdul Aziz, Lorenzo Pellis, Thomas House, Ian Hall

**Affiliations:** ^1^ Department of Mathematics, The University of Manchester, Manchester, UK; ^2^ School of Mathematical Sciences, University of Nottingham, Nottingham, UK; ^3^ COVID-19 Vaccines and Epidemiology, UK Health Security Agency, London, UK

**Keywords:** reproduction number, time-varying transmission, compartmental models, generalized additive models

## Abstract

The reproduction number, the mean number of secondary cases infected by each primary case, gives an indication of the effort required to control the disease. Beyond the well-known *basic* reproduction number, there are two natural extensions, namely the *control* and *effective* reproduction numbers. As behaviour, population immunity and viral characteristics can change with time, these reproduction numbers can vary over time. Real-world data can be complex, so in this work we consider a generalized additive model to smooth surveillance data through the explicit incorporation of day-of-the-week effects, to provide a simple measure of the time-varying growth rate associated with the data. Converting the resulting spline into an estimator for both the control and effective reproduction numbers requires assumptions on a model structure, which we here assume to be a compartmental model. The reproduction numbers calculated are based on both simulated and real-world data, and are compared with estimates from an already existing tool. The derived method for estimating the time-varying reproduction number is effective, efficient and comparable with other methods. It provides a useful alternative approach, which can be included as part of a toolbox of models, that is particularly apt at smoothing out day-of-the-week effects in surveillance.

## 1. Background and introduction

Severe acute respiratory syndrome coronavirus 2 (SARS-CoV-2) is the etiological agent of the coronavirus disease 2019 (COVID-19). The infection was first identified in China in late 2019, and only a few months later cases were found all around the world and a global pandemic was declared [1]. Due to the high transmission, rapid mutation and changing human behaviour, many countries and regions have experienced multiple outbreaks: for instance, the United Kingdom (UK) experienced a second wave in the winter of 2020, partially driven by Alpha variant; the first case of the Delta variant in the UK was found in mid-April of 2021 [2] and generated a new wave that summer; and in December 2021 the Omicron wave began [3]. In such situations, a method for estimation of the transmission rate over time is needed because time-varying factors influencing transmission will impact the effectiveness of the mitigation deployed. Therefore, such estimates can inform if stronger or weaker interventions may be required.

There are two commonly used indicators to assess the transmission impact of an epidemic, namely growth rate and reproduction number. The former describes the rate of change of the observed cases, which provides a statistical measure indicating if the infectious disease risk is increasing (positive) or decreasing (negative) [4,5]. Work focusing on this indicator includes, for example, a recent approach that uses Bayesian modelling with Gaussian processes to estimate the growth rate of SARS-CoV-2 in England [6]. Meanwhile, the reproduction number can be a more useful metric for decision makers as it can inform the extent of public interventions against the disease. For instance, if a proposed intervention was to reduce the infection rate by a factor equalling the reciprocal of reproduction number then this may be expected to be sufficient for an effective control. Hence, obtaining the real-time reproduction number is also helpful to flexibly adjust the intervention policy [4].

The basic reproduction number is defined as the mean number of secondary cases infected by a primary case in an otherwise wholly susceptible population. For the simple compartmental SIR model, it is defined as $R_{0}=\beta /\gamma$, where β is the infection rate (a composite of the average number of contacts and the probability of infection given a contact) and γ is the removal rate [7]. A systematic review [8] has done a full-text assessment of 23 studies estimating the basic reproduction number of SARS-CoV-2 early in the pandemic, obtaining an overall mean of 3.38±1.40, which is consistent with the challenges in controlling its rapid spread [5]. The fact that such a range of different estimates exist is not surprising: $R_{0}$ can virtually never be measured directly, but rather needs to be estimated through the use of a model, and different assumptions on model structure and parameter values might lead to different estimates; $R_{0}$ is not only a property of the virus, but also of the population characteristics and mixing patterns, and so it might naturally vary between different settings, geographical regions and populations; and finally, as it became evident during the COVID-19 pandemic, the intrinsic transmissibility of the virus is also not immutable, as variants might evolve which are more efficient at spreading and can outcompete the previous ones. Even when restricting to a setting and time where it is reasonable to assume a single value of $R_{0}$, individual and societal behavioural responses may change when faced with a disease [9]. Following Pellis *et al.* [4], the *effective* reproduction number $R_{E}(t)$ (often denoted by $R_{t}$) describes the expected number of secondary infections under the current conditions of population mixing, transmission and immunity. The *control* reproduction number (or reproduction number excluding immunity), $R_{C}(t)$, describes the expected number of secondary infections under the current contact and transmission patterns in an otherwise fully susceptible population [4]. The control and effective reproduction numbers can vary with time and are context-specific so driven by the data used in the inference.

Previous work has considered methods to estimate time-varying reproduction numbers. For example, before the COVID-19 pandemic, a study modelled the reproduction number by multiplicative random walk and applied it in stochastic SIR model to explore the Ebola outbreak [10]. Cori *et al.* [11] give an approach to inferring the effective reproduction number over time from case data and information of serial interval (time difference of symptom onset between the infector and the infected individual), and the corresponding R package EpiEstim [12] has made inference of such a time-varying reproduction number accessible to wider user community. Subsequent studies work out the distribution of time-varying reproduction number conditional on serial interval, and surmises the reasons why the reproduction number is time-dependent, including but not necessarily disease control interventions [13], and EpiEstim has been updated to account for imported cases in the reproduction number estimation. Here we use $R_{t}$ estimates from EpiEstim as a comparator for the method developed in this study. However, measuring the serial interval is not always straightforward, as evidenced for example by the variability observable in a systematic review [14].

While this work had begun in 2020 (arising from ideas posited in [5]), we conducted a scoping literature search in December 2023 on PUBMED for recent similar articles. Searching (‘Time varying’ AND ‘Reproduction Number’) yielded 236 returns. Exclusion on title, abstract and full text (not respiratory disease, no explicit mention of time-varying infection rate) gave 38 studies explicitly considering time-varying reproduction number estimates. After a full read, we concluded that the majority of studies (31) had been cited above, directly used EpiEstim (or a variant of this method) or were applying alternative analyses based on renewal equations [15]. The developers of EpiEstim have conducted a literature review of recent applications [16]. In general, these studies hinge on direct measurement of either the generation time or serial interval.

Another issue for modelling the SARS-CoV-2 outbreak is parametrization of the effectiveness of pandemic interventions, because natural and mandated human behaviour change can influence disease transmission, so improper assumptions for behaviour change in the epidemic modelling will yield biases [17]. Modelling with a (semi-parametric) smoothing process can be an alternative option when mechanistic assumptions are unclear or unknown. Two studies from the literature search [18,19] fuse deep learning techniques and Kalman filters, respectively, to compartmental models.

A generalized additive model (GAM) [20,21] is an established statistical tool that can be applied on case data. In this study, we propose a novel technique for inferring the reproduction number using GAMs to derive growth rates and, with structural assumptions on the mechanisms of transmission, to translate them to reproduction number estimates. The remaining five studies, all published since 2020, from the literature review have performed similar analyses. To infer a time-varying reproduction number Hong and Li apply cubic B-splines with Poisson noise [22], Gressani *et al.* extend this with a negative binomial noise model [23], while Eales *et al.* use Bayesian P-splines and verify the result by comparing it with a simple exponential epidemic model [24]. Wood and Wit use adaptive smoothing splines [25] with negative binomial noise and a highly structured model. Gleeson *et al.* employs a negative binomial noise term with a GAM using thin plate splines integrated with a compartmental model [26]. This study is similar in concept to ours, but while the authors have gone into more detail on the criteria for inversion of compartmental models, our approach was to apply this method to social care data and we consider two different compartmental models (as we consider two different datasets from care home surveillance). Furthermore, we have focused here on the verification of our method via simulations as well as with real-world data. Other differences include the fact that the approach of Gleeson *et al.* is fully Bayesian (using the brms package) and that by using the mgcv package [27] we can switch flexibly between splines. We also explore the use of day-of-the-week terms in the regression to account for structured surveillance bias.

## 2. Material and methods

GAMs [20] have been used for rapid estimation of the instantaneous growth rates in support of policy decisions in the United Kingdom [5,28]. However, reproduction number estimates can offer an insight on the amount of transmission that needs to be prevented to control the spread, thus offering additional context to the calculated growth rate, but requires additional structural assumptions to be made. In this study, new estimators of time-varying reproduction numbers are developed by combining GAMs (with time as the argument of the smoothing function) and compartmental transmission models based on ordinary differential equations.

### 2.1. Modelling the daily incidence time series

The daily case incidence data $C_{t}$ is modelled by a GAM with log link assuming negative binomial error structure, so that


(2.1) $$C_{t}∼NegBinom(m(t),\theta );\;\ln m(t)=c+s(t),$$

where t denotes time (throughout, we use day as the time unit), the function $m(t)=E[C_{t}]$ denotes the expected number of cases at time t while c and s(t) are the ‘intercept’ (constant over time) and the smooth function (spline) over time, respectively [20]. The expected value of the spline over time is zero (E[s(t)]=0 over the length of the time series) and so c=E[ln⁡m(t)]. For a generic function f, we use the $f_{t}$ subscript notation to denote surveillance data on day t and f(t) to denote the continuous function we want to fit to such data.

Splines have been commonly used to apply a smoothing process to data. Here we use penalized splines, which involve the use of a roughness penalty, say $\int_{a}^{b}f^{\prime \prime }(z)dz$, instead of the number of basis functions, to control the smoothness of the fitted curve [29]. However, other spline options available in the mgcv package (e.g. cubic spline and Gaussian Process) have been tested without dramatic influence on results (not shown).

Beyond the choice of which splines to use, the choice of knots (basis dimension) has important repercussion on the resulting fitted function, and problems may arise if they are too many or too few. In this paper, we will use a spline with k=floor(K/τ) knots where K is the total number of data points in time series being fitted and τ is indicative of the gap between knots (subject to a lower bound of k=10 if K<10τ). The rationale for this is that shorter gaps between knots may over-fit data and interact with the day of week terms, while longer gaps between knots would under-smooth the data and inhibit detection of changes in trend due to variants and interventions. The choice of τ is predicated by the epidemiological model discussed later.

Fitting a continuous function m(t) to discrete data $C_{t}$ allows us to define an instantaneous growth rate at any time point t, usually denoted by r(t), which can be computed as the per-capita variation in the number of cases (i.e. $\dot{m}(t)/m(t)$). In terms of the elements of the GAM (see also [5]), this is given by


(2.2) $$r(t)=\frac{\dot{m}(t)}{m(t)}=\frac{\dot{s}(t)\exp\left(c+s(t)\right)}{\exp\left(c+s(t)\right)}=\dot{s}(t).$$

The intercept c may contain non-temporal fixed effects or random effects. Regular spikes in surveillance data (periodic every 7 days) (figures 5, top panel, and 6, top panel) are probably due to operational constraints in data reporting and collecting pattern or other systematic lags. Hence the generalized additive model, when fitted to care home datasets, should include a parametric component to account for day-of-the-week effects,


(2.3) $$\ln(m(t))=c+{weekday}_{t}+s(t).$$

Thus when fit to data we will find coefficients for the parametric terms c and categorical variables ${\mathrm{weekday}}_{t}$ and the spline $s(t)=\sum_{j=1}^{k-1}s_{j}(t)\sigma_{j}$ for a set of k−1 unknown coefficients $\sigma_{j}$ and basis functions $s_{j\;}$, where as before k is the number of knots (‘1 degree of freedom is usually lost to the identifiability constraint on the smooth’) [20,27]. In general, then $\ln(m(t))=c+{\mathrm{weekday}}_{t}+\sum_{j=1}^{k-1}s_{j}(t)\sigma_{j}$.

The estimation process will produce the mean and standard deviation given the sample, which define the multivariate-normal distribution of our parameters (both the day-of-the-week terms and the coefficients of the spline). This can be used to generate credible intervals (CrIs) by simulating vectors of parameters from it and multiply them with the design matrix (sometimes called the model matrix, which can be obtained as an optional output from the predict function in the mgcv package) to provide (approximate) estimates of ln⁡(m). However, although the multivariate-normal distribution’s mean and covariance matrix can be simply extracted from model output, they are actually conditional on the fixed smoothing parameters, which have been determined in the model fitting. Therefore, we instead use a Bayesian parametric simulation approach to generate asymptotic unconditional CrIs for the GAM predictions, as described in [20]. In simulation and testing, the conditional CrIs and unconditional CrIs were not very different but the latter is presented in the results below.

### 2.2. Modelling a time-varying infection rate

The force of infection to a susceptible individual in a compartmental model is often denoted βI/N, where β is the constant infection rate per capita and I the total number of infectious people (at time t) in a population of size N. Rather than assuming β is constant we redefine this infection rate as β(t)=νρ(t), such that the time dependency is encoded in the dimensionless function ρ while ν is a time-invariant rate defined such that ρ is a measure of average total infectivity spread by a single individual and that in the early epidemic phase with unconstrained exponential growth it would be equal to the usual threshold parameter $R_{0}$ of an SIR-type compartmental model [4].

As such ρ(t) may be considered as a measure of the control reproduction number. Of course, the disease-specific natural history such as infectious period may vary over time, particularly for extended pandemic periods spanning multiple years, with different variants emerging and different interventions being deployed. Thus ν is better thought of as simply a characteristic scaling factor and ρ gives insights into virological, behaviour and intervention impacts that will potentially require specific interpretation once calculated.

The infection rate β is a function itself of the probability of infection given contact and contact rate [7]. An intervention may appear to act to reduce the infectious period by some fraction—say isolating someone after 2 days—while in the model this effect might be rendered by keeping the infectious period the same but reducing the number of contacts. A variant may change presentation of disease and modify the infectious period (or probability of infection). The impact of variants, therefore, needs caution when interpreting the results but using a single value of ν allows a stable interpretation of the derived ρ values across time.

The function ρ is then unknown *a priori* and our aim is to infer it from the available data, which in this work we consider being either incidence data $C_{t}$ (new observed cases per day) or mortality data $D_{t}$ (new deaths per day). Although the discrete data are technically the integral of the rates at which cases or deaths are observed, for simplicity we assume that the continuous functions fitted are sufficiently smoothly changing that discrete data are well approximated by the rates themselves.

#### 2.2.1. Modelling outbreak declarations in care homes

The SIS model [7] is a simple yet useful model of disease spread, and here is used to model the infectious status of entire care homes, rather than of each of the residents in them. Therefore, a care home with no reported COVID-19-positive residents is considered as a susceptible ‘individual’, which will move to the infected state when the care home detects their first case, and shall shift back to the ‘S’ stage after the outbreak is declared closed [30]. Clearly a care home cannot move to physically infect another setting, but in the early stages of the pandemic staff worked in multiple care homes [31], and even without direct connection staff, visitors and residents interact with the wider community on a daily basis. Therefore the SIS structure is feasible for data showing the number of care homes currently in outbreak status, denoted by I, and those not in outbreak status, denoted by S. Then, given S=N−I, where N is the total number of English care homes (assumed constant on the timescale of a pandemic), a single equation describes the system


(2.4) $$\begin{array}{ccc} & \dot{I}(t)=\beta (t)\frac{S(t)I(t)}{N}-\gamma I(t)=\beta (t)\frac{I(t)(N-I(t))}{N}-\gamma I(t), & \end{array}$$

where β(t) and γ are, respectively, the time-varying infection rate and the removal rate. Equation (2.4) can also be rewritten as


$$\dot{I}(t)=\gamma \left(\rho (t)\frac{\nu (N-I(t))}{\gamma N}-1\right)I(t).$$

Given $R_{0}=\beta /\gamma$ in a standard SIS model, then we set ν=γ and equate the incidence measure from the GAM with the incidence predicted from the SIS model, namely


$$e^{c+s(t)}=\gamma \rho (t)\frac{I(t)(N-I(t))}{N}.$$

Therefore


(2.5) $$\rho (t)=\frac{N\exp(c+s(t))}{\gamma (N-I(t))I(t)}=\frac{\exp(c+s(t))}{\gamma I(t)}\frac{N}{N-I(t)},$$

which is a measure of spread *between* care home, for example through sharing of staff or less direct transmission routes like chain of infection in members of the community. Note that, in the rightmost expression, we have factorized the control reproduction number in a term that represents the effective reproduction number and the term N/S(t), which accounts for the depletion of susceptibles in the population. Replacing the incidence βSI/N in (2.4) with $e^{c+s(t)}$ and treating it as the external source term when applying the variation of parameters method, we obtain


(2.6) $$I(t)=e^{-\gamma t}\left[I(0)+\int_{0}^{t}\exp(c+s(u)+\gamma u)\;du\right]$$

and substitution into (2.5) gives the following estimator of the control reproduction number directly from the time-varying growth rate s(t),


(2.7) $$\rho (t)=\frac{e^{c+s(t)+\gamma t}}{\gamma \left[I(0)+\int_{0}^{t}e^{c+s(u)+\gamma u}\;du\right]}\times \frac{N}{N-e^{-\gamma t}\left[I(0)+\int_{0}^{t}e^{c+s(u)+\gamma u}\;du\right]}.$$

Hence, we may create an estimator of the control reproduction number ρ(t) assuming independent constant estimate values of I(0), γ and N. Thus, while there are three parameter values required for evaluation of the control reproduction number, we may expect I(0) to only be critical on short time series of data relative to the duration of a within-care-home outbreak (note that in (2.7) I(0) is scaled by a factor $e^{-\gamma t}$ coming from the numerator, so as t→∞ the contribution of I(0) becomes increasingly small—as long as s(t) is not negative and too large in absolute value; see further comments on this in the electronic supplementary material, section 1). The parameter N is fixed as the observed number of care homes in England and is derived from the Care Quality Commission. Hence the parameter γ is probably the most critical and sensitive one. We can take γ=1/T where T is the average duration of an outbreak. In this case, for a point estimate of ρ(t) at a fixed time t we may sample estimates of c and s(t) from surveillance data and include uncertainty on T by sampling from the distribution on mean outbreak duration assuming N is fixed and I(0) has burnt off. For simplicity, however, in this work we fix T so CrIs reflect only the uncertainty from the GAM fit to surveillance data. Furthermore, we choose τ=T to fix the gap between knots to be the recovery time of homes from outbreaks (equivalent for SIS model to the generation time).

The effective reproduction number, denoted $R_{E}(t)$, in this case is


$$R_{E}(t)=\gamma^{-1}{\left[I(0)e^{-(c+s(t)+\gamma t)}+\int_{0}^{t}e^{s(u)-s(t)+\gamma (u-t)}\;du\right]}^{-1},$$

which is obtained from (2.7) by simply writing the first term in an alternative form and disregarding the second term, which captures the depletion of susceptibles. Note that, in a large population with low initial prevalence, the depletion of susceptible is negligible for some time, so control and effective reproduction numbers are approximately equal, i.e. $\rho (t)\approx R_{E}(t)$. In the electronic supplementary material, section 1, we show that, in this case, an even simpler approximation to both reproduction numbers $\rho^{*}(t)=R_{E}^{*}(t)=1+\dot{s}(t)/\gamma$ can be derived when assuming any transients arising from the initial conditions have passed and s(t) is approximately linear, i.e. the incidence is growing or declining approximately exponentially, for some time.

#### 2.2.2. Modelling individual cases across care home settings

For data measuring case incidence, an SEIR model structure is assumed as a representative approximation to the disease dynamics. The standard SEIR model might need some adaptation for application to a residential setting (such as care home or prison) as some of the residents might pass away or leave due to non-COVID reasons, and then the arising vacated rooms would be filled by new residents. For simplicity, we modelled this replacement as instantaneous (though in reality there may be a period of vacancy), but we allow new individuals to be either susceptible or immune (e.g. both due to natural infection or vaccination). Therefore, we have


$$\begin{array}{rl} \dot{S}(t) & =-\beta (t)\frac{S(t)I(t)}{N}-\mu S(t)+(1-\theta )(\mu N+\delta \gamma I(t))\\ \dot{E}(t) & =\beta (t)\frac{S(t)I(t)}{N}-\alpha E(t)-\mu E(t)\\ \dot{I}(t) & =\alpha E(t)-\gamma I(t)-\mu I(t)\\ \dot{R}(t) & =(1-\delta )\gamma I(t)-\mu R(t)+\theta (\mu N+\delta \gamma I(t))\\ \dot{D}(t) & =\delta \gamma I(t), \end{array}$$

where disease states S, E, I, R and D denote the numbers of susceptible, exposed, infected, recovered and deceased, respectively, β(t) and γ are (as before), the infection and removal rate, respectively, α is the rate of becoming infectious, δ is the COVID-19-specific case fatality ratio, μ is the rate at which residents leave the care home (natural mortality rate) and θ is the proportion of new entries that have immunity to the infection (this is not necessarily the same as conferred immunity in residents and staff from past outbreaks). An individual leaving the population is assumed to be immediately replaced by a new individual (immune with probability θ or otherwise susceptible), so the total population N=S+E+I+R (i.e. the sum of those in living state) is constant, and D is effectively only used to keep track of the number of COVID-19 deaths. In the use case that the surveillance data does not permit tracking the population turnover due to natural mortality, μ may be set to zero. Note that θ will in general vary with time due to vaccination policy or community epidemics, but at the start of a new pandemic we expect θ=0.

To derive an estimator for the time-varying reproduction number ρ(t) we substitute β(t)=νρ(t). In this model, disease incidence (new entrants to I state) is given by $\alpha E(t)=e^{c+s(t)}$. Solving for E,I and R means that


(2.8) $$\begin{array}{rl} E(t) & \;=\;\frac{1}{\alpha }e^{c+s(t)}\\ I(t) & \;=\;e^{-(\gamma +\mu )t}\left(I(0)+\int_{0}^{t}\exp\left(c+s(u)+(\gamma +\mu )u\right)\;du\right)\\ R(t) & \;=\;e^{-\mu t}\left(R(0)+\theta N(e^{\mu t}-1)+(1-\delta +\delta \theta )\gamma \int_{0}^{t}I(u)e^{\mu u}\;du\right). \end{array}$$

We are thus assuming that $E(0)=e^{c+s(0)}/\alpha$; other initial conditions (I(0) and R(0)) are discussed below.

Because individuals can leave the care homes due to natural mortality, the average time length of any infected individual being infectious is 1/(γ+μ) rather than 1/γ, and individuals leave the E stage at the rate of α+μ but the rate of entering the I stage is α. Therefore, only a fraction α/(α+μ) of the infected individuals can become infectious, with the opposite fraction dying while in the E state. Thus, in this model the critical scaling timescale on the reproduction number is


(2.9) $$\begin{array}{ccc} & \nu =\frac{\left(\gamma +\mu )(\alpha +\mu \right)}{\alpha }. & \end{array}$$

The control reproduction number estimator is given by


(2.10) $$\begin{array}{rl} \rho (t)= & \frac{\dot{s}(t)+\alpha +\mu }{\left(\alpha +\mu )(\gamma +\mu \right)}\times \frac{\exp\left(c+s(t)+(\gamma +\mu )t\right)}{I(0)+\int_{0}^{t}\exp\left(c+s(u)+(\gamma +\mu )u\right)\;du}\times \frac{N}{N-E(t)-I(t)-R(t)}, \end{array}$$

where E(t),I(t) and R(t) are as in (2.8). The estimator ρ(t) may then be inferred from surveillance data for independent estimates of parameters γ, α, μ, δ, θ, I(0), R(0) and N. As in the previous section, the initial conditions (I(0) and, if μ>0, R(0)) have only transient effects and population size N can be learnt from the surveillance data being used. In this study, we assume for simplicity low community immunity and limited community vaccination, and hence θ=0. However, future work will consider the impact of vaccination (community and setting specific) and a time-varying θ on calculation of the control reproduction number. In §3.3, we will also further assume for simplicity no natural or disease-induced mortality (μ=δ=0, but for a case where δ>0 see the electronic supplementary material, section 2). With these parameter choices the value of R(0) can have significant impact on the obtained estimates: our choice is to fix it at zero given the context of a pandemic of a new infectious disease, but this may not always be the case. The impact of the choice of I(0), instead, fades out rapidly after the initial transients. The disease natural history parameters require independent estimates and we fix τ=2(1/α+1/γ). The choice of multiplying by 2 is made because the generation time 1/α+1/γ is relatively short compared with the duration of the time series.

The effective reproduction number is then obtained when the last factor N/S in (2.10) is removed,


$$R_{E}(t)=\frac{\dot{s}(t)+\alpha +\mu }{\left(\alpha +\mu )(\gamma +\mu \right)}\times \frac{\exp\left(c+s(t)+(\gamma +\mu )t\right)}{I(0)+\int_{0}^{t}\exp\left(c+s(u)+(\gamma +\mu )u\right)\;du}.$$

When the population is almost entirely susceptible (prevalence, incidence and θ are small), the effective and control reproduction numbers are approximately the same. In this case, with similar arguments as in the electronic supplementary material, section 1, assuming the transient has burnt out, that replenishment is slower relative to disease transit rates (μ is negligible compared with γ), and that the incidence is growing approximately exponentially for a while, we may derive a similar approximation to that for the SIS model (compare with [15]),


$$\rho^{*}(t)=R_{E}^{*}(t)=\left(\frac{\dot{s}(t)+\alpha }{\alpha }\right)\left(\frac{\dot{s}(t)+\gamma }{\gamma }\right)=\left(1+\frac{\dot{s}(t)}{\alpha }\right)\left(1+\frac{\dot{s}(t)}{\gamma }\right).$$

The methods presented here can be adapted to consider mortality data rather than case data: an application of this model version to simulated data is provided in the electronic supplementary material, section 2.

### 2.3. Calibration on simulated data

To check if the estimators derived above are appropriate and useful, a first test is to apply them to stochastically simulated data from the epidemic models described above. Keeling and Rohani [7] illustrate the application of the Gillespie algorithm to such epidemic models [32]. Event-based algorithms are typically slow and so a ‘τ-leap’ method [33] is used here instead.

**Figure 1.**
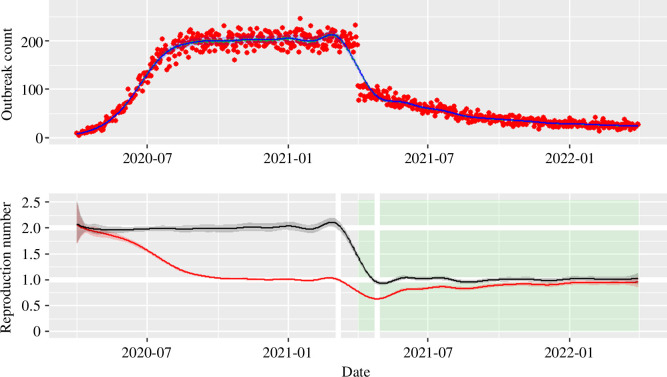
Top: stochastic simulation (τ-leap) for the SIS model. Parameter values are: $N=10^{4},I(0)=100,\gamma =1/25$ with time step of Δt=0.1. Simulation is assumed to start on 1 April 2020 and last for 2 years. The basic reproduction number is $R_{0}=2=\beta /\gamma$ , and then after 1 year an intervention is implemented reducing transmission by a half (so $R_{C}=1$ from this point). Red points represent the simulated incidence data and the blue line the resulting GAM fit, assuming a negative binomial family. Bottom: estimated control reproduction number ρ(t) (black solid line) and effective reproduction number $R_{E}(t)$ (red solid line) from the simulated data shown in the top panel. The shaded ribbons around both central estimates are the sampled 95% CrI. The period with intervention in place is depicted by the green area. White horizontal lines show the reproduction number in simulation before and after simulation and white vertical lines show the date the intervention is applied ±1 generation (here 25 days).

The SIS simulation (figure 1, top panel, showing the arising incidence from the SIS model) is initiated with population N=10000, I(0)=100 and γ=1/25 (25 days for recovery on average), all three chosen as ‘round’ numbers indicative of the number of care homes and duration of outbreaks, with a time step of Δt=0.1 days for a simulation of 2 years starting 1 April 2020. These choices are not particularly crucial as the generation time simply scales the speed of incidence reaching a plateau, the overall population controls the absolute magnitude of that plateau, and the start date is arbitrary. The transmission rate is taken to be β=0.1 for dates before 1 April 2021 (1 year after the initial cases seeded the epidemic). This translates to a reproduction number of $R_{0}=R_{C}(t)=2$ for t<01/04/2021. The control reproduction number drops post-intervention to $R_{C}=1$. Incidence is given by βI(1−I/N) and *without intervention* we expect from standard theory that $\lim_{t\to \infty }I(t)=N(1-1/R_{0})$ and hence an incidence of $\beta N(1-1/R_{0})(1/R_{0})=200$, which the simulation attains from August 2020 to time of the intervention. The post-intervention value of $R_{C}$ is chosen to reach the value of 1 and so incidence will eventually drop to zero. The intervention is only just sufficient to eventually succeed in control.

**Figure 2.**
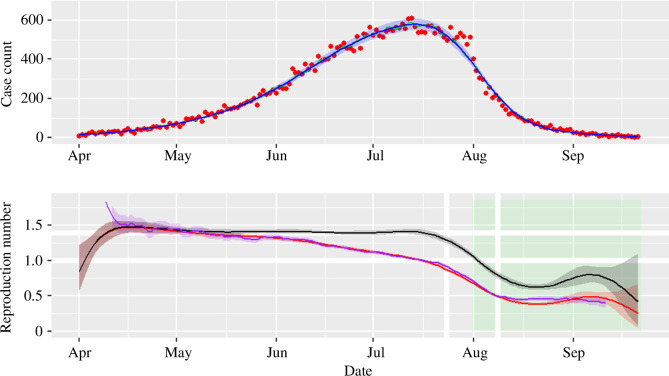
Top: stochastic simulation (τ-leap) for the SEIR model. Parameter values are: $N=10^{5},E(0)=0,I(0)=100,\alpha =1/3,\gamma =1/5$ with time step for leap of Δt=0.1. The basic reproduction number is set to infect 50% of population without intervention ($R_{0}=\beta /\gamma =2\;\log\;2\approx 1.39$). The epidemic is assumed to start on 1 April 2020 and there is an intervention implemented on 31 July 2020 that immediately reduces $R_{0}$ by half (so $R_{C}=\log(2)\approx 0.69$ from that date onwards). Red points represent the simulated incidence data from SEIR model and the blue line the resulting GAM fit, assuming negative binomial distribution family. Bottom: estimated reproduction number ρ(t) (black solid line) from the simulated data from the SEIR model (with the 95% CrI depicted by a grey shade ribbon). The red line shows the estimate of the effective reproduction number $R_{E}$ (with the 95% CrI depicted by the red shaded region) with EpiEstim estimates (purple) also shown for comparison. Horizontal white reference lines mark the value of $R_{0}=2\;\log\;2$ and $R_{0}=1$. The green shade marks the period under intervention and white vertical lines show date the intervention is applied ±1 generation (in this case 8 days).

The simulated incidence from an SEIR model (figure 2, top panel) is initialized with 100 infected and 0 exposed individuals among a total population of 100000. The average incubation period is 3 days (α=1/3), the average infectious period is 5 days (γ=1/5), and the time step used in the simulation is Δt=0.1 days. For simplicity, we set the rate at which residents leave care home μ=0, the community immunity θ=0 and the case fatality ratio δ=0. To model an intervention after four months (31 July 2020), the infection rate is defined as β=0.4log⁡2 before intervention (so $R_{0}=R_{C}=2\;\log\;2\approx 1.39$, which would eventually with no intervention infect 50% of population). After the intervention $R_{C}=\log\;2\approx 0.69$, which is less than 1, and so control should be achieved. There is no imputed reporting bias by weekday. The incidence peaks close to the time of the intervention but the peak is due to depletion of susceptibles rather than the intervention.

### 2.4. Data

Having tested that the method is able to recover known parameters from simulated data, the method is then applied to real-world datasets.

All positive COVID-19 tests were asked for the residential address, though when not available the GP-registered address was used. Within UK Health Security Agency (UKHSA), address information was provided by the Second Generation Surveillance System (SGSS) [34] and the UKHSA Geospatial Team then matched addresses to the Ordnance Survey database to obtain a Unique Property Reference Number (UPRN) and Basic Land Property Unit (BLPU) class information.

Addresses with BLPU classes of RI01 were identified as care homes. Care Quality Commission (CQC) IDs for care homes and their accompanying UPRN information were also linked to the cases line list to identify registered care providers not identified through the address matching process. Finally, properties with ‘care home’, ‘rest home’, ‘senior living’, ‘elderly’, ‘retirement’ were also searched to determine whether there were any additional addresses that might have been care homes [35,36]. The total number of care homes for older people is approximately 14 500, with a total of approximately 450 000 beds associated with these settings [37]. We use the number of beds as a proxy for the population size. We expect this to be an overestimate as care homes typically operate at 90% capacity, but a precise value of the number of residents is hard to specify and probably fluctuates significantly over time.

This linked dataset was used to create two aggregated and non-identifiable samples, namely:

1. Outbreak declaration data: the number of care homes in England that declared an outbreak on given days from 01/04/2020 to 06/10/2021.
2. Positive-test data: all cases attributed to adult social care settings were aggregated by day of positive test to give the number of new confirmed cases among care home residents in England from 15/03/2020 to 09/10/2021.

The estimated time-varying reproduction numbers inferred are relevant to the dataset (and underlying population) in question and may differ from published national estimates for other settings or communities. Furthermore we do not consider different risk group reproduction numbers, as this would require, for example, separate data on residents and staff in homes. Such structured analysis is not possible with current data and is subject to future research. As such, the estimated reproduction number can be considered indicative of the (additional) control effort required at that time but may not formally translate to point estimates of transmission from specific settings.

## 3. Results

### 3.1. Simulation results

The reproduction number estimators from the SIS simulation are shown in figure 1 (bottom panel). The estimation is mostly accurate. There is a period before and after the intervention with similar deviation from the true value. The effective reproduction number estimator (figure 1, bottom panel: red line) behaves as we might expect, and as prevalence increases the estimate of $R_{E}$ diverges from that of $R_{C}$. When the incidence plateaus after August but before the intervention, the effective reproduction number is 1.

To discuss key features in estimators, the concepts of transient and adjustment periods are defined as follows:

— Transient period: the period, at the beginning of the simulation, during which the CrI does not include the pre-intervention $R_{0}$.
— Adjustment period: the period from the time the upper CrI stops including the pre-intervention $R_{0}$ to the time when the lower bound firstly includes the post intervention $R_{0}$.

It may be possible for either or both time periods to have length 0 in some simulations.

**Figure 3.**
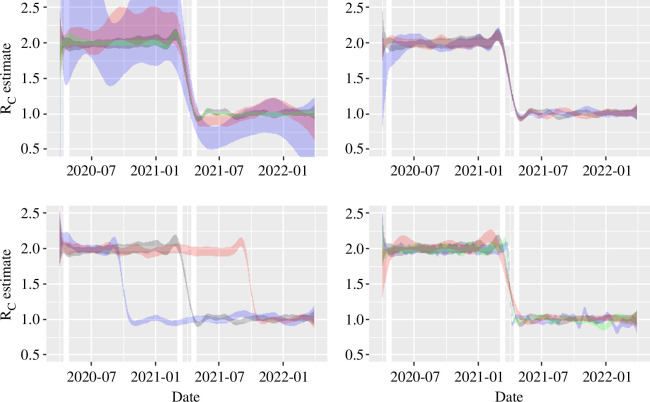
Control reproduction number estimates from a stochastic SIS model. Ribbons in each panel show the Bayesian CrI generated using the conditional simulation method in [20]. Unless otherwise stated the baseline parameters in epidemic model are $N=10^{4}$, $I_{0}=10^{2}$, γ=1/25 with intervention occurring on 1 April 2021 (1 year after introduction). Top left: variation in estimator by population size: $N=10^{2}$ blue, $N=10^{3}$ red, $N=10^{4}$ grey, $N=10^{5}$ green; top right: variation in estimator by initial condition: $I_{0}=10$ blue, $I_{0}=100$ grey, $I_{0}=1000$ red; bottom left: variation in estimator by intervention date: 1 April 2021 grey, 1 October 2020 blue, 1 October 2021 red; bottom right: variation in estimator by generation time: 50 days red, 25 days grey, 10 days green, 5 days blue. Thick white vertical lines are reference lines at ± one generation times from the date of intervention (though the equivalent intervention time guides for earlier and later simulations in the bottom left panel are not shown for image clarity) or from the first case .

The transient period is not evident for the SIS simulation (see figure 1, bottom panel). We have used in inference the actual initial condition, but if we instead use a proxy estimate of the initial conditions (say $I_{0}=C_{0}/\gamma$, where $C_{0}$ is the initial observed incidence data point) then we observe a transient period arise as an artefact of stochastic noise in simulation. In figure 3, we see little evidence of *sustained* appearance of a transient period for the SIS-based inference, unless the initial seeding of the outbreak is small relative to the total population (see the blue ribbon in the top right panel) due to greater stochastic variation. However, when the transient does occur, it ‘burns off’ on the timescale of the generation time.

As might be expected, the adjustment period is more salient. This tends to last for the 25 days either side of intervention date as shown in figure 1 by white vertical reference lines. Given the definition above, the adjustment period is a little shorter than this 50-day period but there is a kink above and below the true value. This kink is an artefact of the spline (though remains with other splines considered in the sensitivity analysis, such as Gaussian process splines) and is affected by the choice of τ (or the number of knots).

In the SIS model, we assume the number of knots is ‘duration of outbreak/recovery time/generation time’ rounded down to nearest integer, which in this case is floor(720/25)=28, and if we increase the number of knots used in the GAM then this kink also becomes more visible. This is a sign of the GAM smoother struggling to adapt to a ‘shock’ (i.e. instantaneous) change in transmission (as seen in the top panel of figure 1). We deliberately model the intervention as a shock to stress-test the inference. Despite this, the adjustment phase tends to be centred on the change timing. While ‘lockdown’ events are rapid there may be a phased transition as people adjust to changes (or uptake is adopted across the country) and for events like vaccination the roll-out will be over a period of time and so in reality this will probably be less of an issue.

In figure 3, we see that this adjustment period is not particularly sensitive to the population size (top left panel), though small at-risk populations carry greater stochastic uncertainty, and changing the initial condition has no meaningful impact on it (top right panel). The bottom left panel shows that the timing of intervention does not affect the adjustment period either.

The generation time does have an effect on the adjustment period (figure 3, bottom right) with longer recovery times (red ribbon, 50 days) more prolonged relative to 25-day or 5-day recovery. This is to be expected as the number of knots being used in the smoother is changing. In addition, longer recoveries tend to exhibit both a more pronounced kink, and a higher variability (i.e. a broader ribbon). While the former is predominantly due to the larger number of knots, the latter is due to a lower incidence at the steady state. For example, a generation time of 50 days (red ribbon) corresponds to a steady state of 100 cases per day, rather than the 200 visible in figure 1 where recovery is 25 days. At the other extreme, a generation time of 5 days (blue ribbon) is more certain, because incidence levels are approximately 1000 cases per day, but the chosen value of τ=5 is over-fitting the time series (suggesting that if the real-world situation being modelled was SIS with a fast recovery the method may need a larger value of τ).

**Figure 4.**
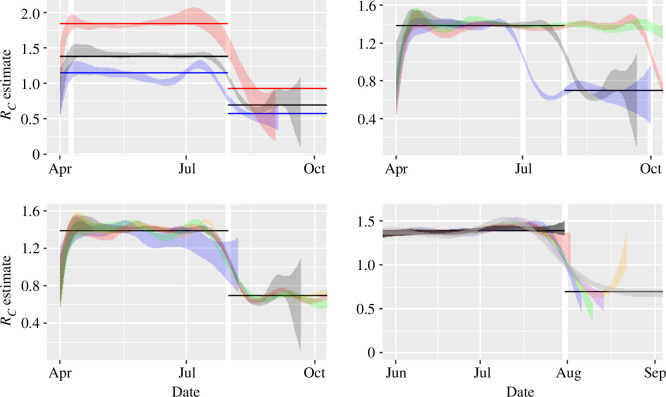
Control reproduction number estimates arising from the stochastic SEIR simulations. The 95% CrI arising from a parametric simulation of SEIR is plotted in each ribbon. Top left: variation by basic reproduction number (red ribbon $R_{0}=(4/3)\;\log\;4$, grey ribbon $R_{0}=2\;\log\;2$ and blue ribbon $R_{0}=4\;\log\;\left(4/3\right)$, corresponding to a final size of 25%, 50% and 75%, respectively); top right: variation by timing of intervention (1 July blue, 31 July grey, 30 Sept red, no intervention green); bottom left: variation by population (blue, population $10^{4}$, grey $10^{5}$, green $4.5\times 10^{5}$, red $10^{6}$ and orange $6\times 10^{7}$); bottom right: variation by subset of sample (see text for explanation, and notice the change in the *x*-axis to zoom in on the three months prior to the intervention). In all plots, the incubation period is 3 days and infectious period is 5 days. The thick white vertical lines show the timing of interventions except in top left panel where the left vertical thick line shows one generation time from start of the simulation and the horizontal lines show the pre- and post-intervention control reproduction numbers used in simulations, with matching colours when needed.

The estimation results for the SEIR simulations (figure 2, bottom panel) follow a similar pattern to the results obtained from the SIS cases. The outbreak simulated in this figure lasted approximately six months. The transient period is evident here and more often present in other simulations (cf. figure 4). This is probably a consequence of the additional latent period (i.e. the class E), which creates some transient dynamics determined by the initial conditions both in the generation of the data in figures 2 and 4 and potentially in choices of initial conditions in the estimation procedure in figure 6 that might be somewhat misaligned with the data, but is typically resolved within a generation time. The E and I compartment numbers drop to relatively low levels (similar to that of the epidemic initial conditions) by the end of the simulation presented. Extending this timeframe with an even lower incidence leads to increasing estimation uncertainty (not shown).

**Figure 5.**
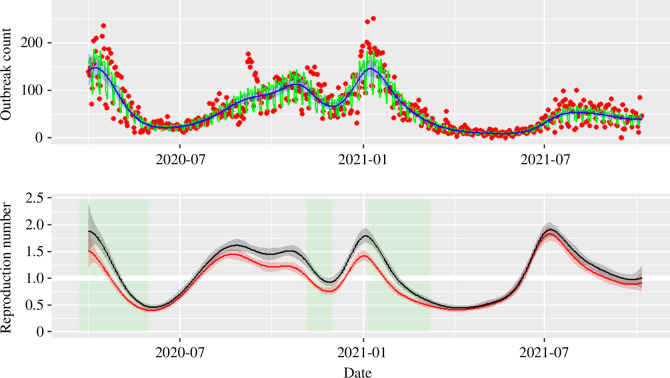
Top: GAM results with options family = nb(link = log), bs = ‘ps’ and k = 21 knots for data on English care homes experiencing an outbreak from 01/04/2020 to 06/10/2021. The data (red points) shows oscillation every 7 days, which are captured as day-of-the-week (DW) effects and interpreted by parametric coefficients (see table 1 for estimates) of the GAM fit (green line). The DW effects can be removed (blue line with 95% CrI shown by blue shaded region) by taking an average of the seven values. Bottom: mean estimate (line) and 95% CrI (shaded band) for the control reproduction number ρ(t) (black) and the effective reproduction number $R_{E}(t)$ (red) for the English care home outbreak data (SIS model structure with N=14500, I(0)=2000, γ=1/26). Both reproduction numbers are generally observed to decreases during lockdowns (green shaded bands), at least before widespread vaccination in care homes at the start of 2021. A thick white horizontal line is added to highlight the threshold value of 1.

**Figure 6.**
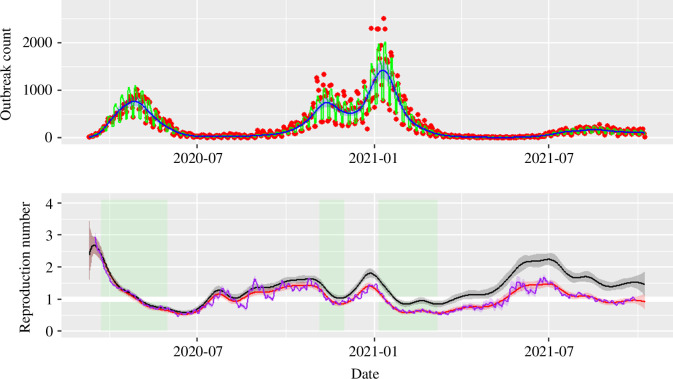
Top: GAM results with options family = nb (link = log), bs = ‘ps’ and k = 35 for the case data recorded in English care homes from 15/03/2020 to 09/10/2021. The data (red points) show spikes every 7 days, which are captured as day-of-the-week (DW) effects and interpreted by the parametric coefficients (see table 1 for estimates) of the GAM fit (green line). The DW effects can be removed (blue line) by taking an average of the seven values. Bottom: mean estimate (line) and 95% CrI (shaded band) for ρ(t) (black) and $R_{E}(t)$ (red) for the model fitted to case data from English care homes (SEIR structure with N=450000, I(0)=500, R(0)=0, infectious period 5 days, incubation period 3 days). The mean estimate (purple line) and 95% CrI (purple shaded band) from EpiEstim (with serial interval following a Gamma distribution with mean 8 and standard deviation 5.83 days) follows the general scale and trend of the effective reproduction number estimated with the method proposed here. A thicker horizontal white line is added to highlight when the estimated reproduction numbers cross the unity threshold.

As with the SIS model, the estimated control reproduction number decreases around the sudden change in transmission (figure 2, bottom panel, note the shock on transmission is not immediately seen in incidence (top panel) due to the presence of the incubation period). The white vertical lines mark ± one generation time (in this case approximated by the sum of the infectious and incubation periods, totalling 8 days). In the SEIR case, we use a number of knots in the GAM so that knots are spaced two generations apart (in this case then the simulation lasted 174 days so 10 knots were used). To try to explain the data given this constraint the GAM slightly under-fits around the intervention and so the control reproduction number estimate in late August is underestimated. This amount of over-/under-fitting either side of the intervention time is subject to variation in simulations (not formally shown but an indication is visible in the panels in figure 4).

Figure 4 shows the impact of different epidemic model parameter assumptions on the derived estimator. The top left panel shows the change in the basic reproduction number. Here we choose an $R_{0}$ that leads to a final size of 25% (blue—$R_{0}=4\;\log\;\left(4/3\right)\approx 1.15$), 50% (grey—the baseline scenario presented in figure 2, $R_{0}=2\;\log\;2\approx 1.39$) and 75% (red—$R_{0}=(4/3)\;\log\;4\approx 1.85$) of a population of 100 000 people. The horizontal coloured lines of matching colour denote the true value used in simulation. Lower values of $R_{0}$ tend to lead to more uncertain estimates early on, due to fewer daily case numbers arising. More specifically, each ribbon becomes narrower just after the peak in incidence (more precisely, at the time of the peak in prevalence). This happens for the red ribbon around May, but then the ribbon widens again in August because the outbreak is effectively over by the time of the intervention due to depletion of susceptibles. The blue ribbon meanders away from the true value before intervention, an artefact of the increased stochastic noise associated with small simulated numbers, but the meandering is centred on the true value.

The top right panel shows the impact of changing the time of intervention (blue: 1 July, grey: 31 July, red: 30 Sept, green: no intervention; these dates are shown with the thick white vertical lines, though the pre- and post-intervention reproduction numbers are shown in black only for the baseline case of the intervention on the 31 July). The general pattern of red and grey is similar and these interventions occur after the natural peak of infections (i.e. the one expected by depletion of the susceptible population). The blue ribbon shows a faster adjustment phase (with intervention occurring before the incidence peak).

The bottom left panel shows the impact on the estimator of changing the population (blue, population $10^{4}$, grey $10^{5}$, green $4.5\times 10^{5}$, red $10^{6}$ and orange $6\times 10^{7}$). The smallest population (blue) has the broadest ribbon but the intervention occurs too late to have a major impact on case numbers. The simulations in the other, larger, populations mean that the intervention is occurring during the period of exponential growth, so the estimates are less uncertain.

The fits to these simulations show varying degrees of oscillation, an artefact of fitting an entire time series with the given number of knots and effectively treating the data as complete and conducting a retrospective analysis. The final panel (bottom right) in figure 4 shows an attempt to investigate the baseline simulation in a real-time ‘mode’ (note the *x*-axis has changed here to zoom in on the three-month period near intervention). The dark grey ribbon shows the 95% CrI for data up to 31 July (the day of the intervention): unsurprisingly, this results in a relatively constant ribbon after the transient period. The red, blue, green, purple and orange ribbons show the results of using 2, 6, 10, 14 and 22 more days of data, respectively, and finally the light grey ribbon shows result from the fit to the entire time series. This shows that evaluating the estimate of $R_{C}$ as the data arrives in real time should be treated with caution at this stage of development. Uncertainty is greatest from the GAM at the extremes of the time series and variation in estimates (due to ‘kinks’ in the spline) for most recent times must be interpreted cautiously and possibly integrated with other contextual information from the outbreak. This requires further simulations and testing.

### 3.2. SIS: outbreak data

Stable reporting of outbreaks started in April 2020. According to the outbreak data, approximately 150 to 200 care homes per day started experiencing an outbreak during April 2020, although this number then dropped in the following three months. Numbers increased from July 2020, until the second national lockdown in November 2020. However, a higher peak at 250 was reached during the Christmas break, followed by a dramatic decrease during the period of the third lockdown and the start of the vaccine campaign from January to March 2021. The spread of SARS-CoV-2 in the English care homes then apparently eased for two months until June 2021. After that, a new wave of infection then took place again when the ‘Delta’ variant started to spread around the country, although the daily counts stayed mostly under 100 (figure 5, top panel, black line).

The outbreak data show periodic weekly fluctuations, indicating the presence of day-of-the-week effects in the reporting mechanism. Therefore, the GAM fitted to outbreak data should include a parametric term for day-of-the-week effects (see equation (2.3)). The smoother uses the penalized spline, and the number of knots is set to 21 for 554 days in total (i.e. the arguments in the spline function in R are set to: bs = ‘ps’, k = floor (554/26)).

The coefficients of intercept and factors (weekdays) are summarized in table 1, referring to ‘Friday’ as the baseline. Overall, Monday usually sees the most reported outbreaks, while Tuesday and Wednesday’s amount are marginally lower than Friday and Thursday is very similar (the p-value for the effect on these days is not significant at a 5% level). At the weekend, the average count of reported outbreaks are roughly half of Friday’s. Therefore taking arithmetic mean of the coefficients of ‘weekday’, with ‘Friday’ as 0, can help remove the ‘weekly noise’ and produce a smooth representative average curve (figure 5, top panel, blue line) for integration in the compartmental model.

**Table 1.** Parametric coefficients of the GAMs based on care homes experiencing outbreaks (SIS model) and case data (SEIR model). s.e. is the standard error of the associated coefficient estimate (denoted coef), the p column shows the resulting p-value and exp⁡(⋅) shows the coefficient estimate on the original scale of the data. The intercept row shows the estimate for a Friday and then the other rows show the relative difference for each day. In the p-value column, ‘∗∗∗’ represents coefficients with p<0.001 (and so are highly significant factors in the model).

	care home outbreaks (SIS)^a^			cases in care homes (SEIR)^b^		
	coef (s.e.)	p	exp⁡(⋅)	coef (s.e.)	p	exp⁡(⋅)
(intercept)	3.979 (0.033)	∗⁣∗⁣∗	53.5	4.855 (0.032)	∗⁣∗⁣∗	128.3
Monday	0.079 (0.047)	0.09	1.08	0.239 (0.045)	∗⁣∗⁣∗	1.27
Tuesday	−0.091 (0.047)	0.055	0.91	0.286 (0.045)	∗⁣∗⁣∗	1.33
Wednesday	−0.053 (0.047)	0.26	0.95	0.166 (0.045)	∗⁣∗⁣∗	1.18
Thursday	−0.001 (0.047)	0.98	1	0.049 (0.045)	0.273	1.05
Friday	0 (reference)			0 (reference)		
Saturday	−0.472 (0.048)	∗⁣∗⁣∗	0.62	−0.648 (0.046)	∗⁣∗⁣∗	0.52
Sunday	−0.618 (0.049)	∗⁣∗⁣∗	0.53	−0.687 (0.047)	∗⁣∗⁣∗	0.50

^a^ Outbreak model: adjusted R-squared = 0.854, deviance explained = 87.6%, 554 observations, smoothing parameter = 14.0241 estimated by restricted maximum likelihood (REML = −2286.3).

^b^ Case model: adjusted R-squared = 0.92, deviance explained = 95.5%, 574 observations, smoothing parameter = 6.7798 estimated by restricted maximum likelihood (REML = −2937.3).

In figure 5, bottom panel, the green shaded bands stand for the three main lockdowns that aimed at reducing the disease transmission nationally and therefore reduced the interaction between different care homes and between care homes and the community. Before March 2021, ρ decreased during the lockdown periods and then increased during the summer period when restrictions relaxed, indicating that the governmental intervention of reducing social community contacts has probably been helpful for care homes’ pandemic impact reduction. We note that testing became more widespread from late Summer 2020, so the sharp increase during August may be an artefact of historical cases being detected from polymerase chain reaction (PCR, which tests for RNA rather than live virus). Vaccination was introduced into social care settings in December 2020, becoming effective in January 2021, and so the long period of low transmission through spring 2021 is an artefact of the vaccine protecting these settings. The increased transmission in July 2021 was contemporaneous to a national community-level increase but was not sustained.

### 3.3. SEIR: case incidence data

The case incidence data (the number of new individuals testing positive to infection) is taken as a proxy for the number of daily new residents becoming infectious in care homes throughout England. The timeline for these case data is 15/3/2020 to 09/10/2021 (figure 6, top panel), with a similar curve trend as that observed for the outbreak data above (figure 5, top panel). Although this time series covers approximately a year and a half, which is roughly the same as the average lifespan in care homes due to natural mortality, for simplicity and to highlight the difference between control and effective reproduction number, we assume negligible population turnover due to both natural and disease-induced mortality and negligible immunity in the population in the model in §2.2.2 (i.e. μ=δ=θ=0—see instead electronic supplementary material, section 2, for a model with δ>0), thus effectively turning it into a standard SEIR model. The first epidemic peak of care home cases happens at the end of April, with approximately 1000 diagnoses on some days, and a double peak between [28] and February 2021 reaching values of approximately 1400 and 2500, respectively. After the release of the third lockdown, a much smaller sized epidemic is seen when the new variant ‘Delta’ appeared in mid-2021 (figure 6, top panel, red data points).

As with the outbreak data, the case data display day-of-the-week effects. The case data is fitted with a GAM (2.3) with penalized spline as smooth term and 35 knots for 574 days in total (bs = ‘ps’, k = floor (574/16)), and the fitting results are presented as green (with day-of-the-week effects) and blue (averaged over day-of-the-week effects) lines in figure 6 (top panel). The parametric coefficients are summarized in table 1, with Friday as the baseline for the day-of-the-week effects. During the week, the numbers of cases for Monday, Tuesday and Wednesday are significantly higher than that for Friday, while the newly positive case counts on Thursday tend to be close to the amount reported on Friday. Weekends are expected to have fewer positive test results, with both Saturday and Sunday having approximately 50% of Friday’s cases. This probably reflects the average operational timing of regular testing in such settings.

Figure 6 (bottom panel) shows the reproduction number and its CrI after averaging the day-of-the-week effects. The results have a similar pattern as seen in figure 5 (bottom panel), with the three national lockdowns corresponding to periods of reducing values of the reproduction numbers. The control reproduction number (black line and dark grey shaded band) reaches values higher than 2.5 in March 2020 (a value potentially still dependent on our choice of the initial conditions, rather than fully driven by the data), increases from 1 to 1.5 during summer/autumn of 2020, and peaks at a value close to 2 in late December 2020. From December 2020 care homes received the vaccine and so the fast change from ρ=2 to ρ<1 is probably a combination of community lockdown and vaccine protection within settings. In 2021, the reproduction number peaks between June and July, when the ‘Delta’ variant was the dominant variant in England [38].

### 3.4. Comparing with EpiEstim

The EpiEstim R package has been developed to compute the effective reproduction number by combining the incidence data with information from the serial interval, which can either be assumed to follow a plausible prior or be directly obtained from some surveillance system [11,13]. We compare EpiEstim with the results obtained with the method presented here when both are applied to the case data from English care homes (figure 6, bottom panel, purple and pink lines and shaded bands). In the parametrization of the SEIR model the average incubation period is set to 3 days and the average infectious period to 5 days. These are plausible values for SARS-CoV-2 (e.g. in line with [39]) and are taken at face value (i.e. in particular, without any uncertainty) given we are only seeking an indicative parametrization to illustrate the methodology rather than focusing on a precise characterization of the timing of disease progression. For this choice of parameters, the generation time distribution is right-skewed, with mean 8 days and standard deviation of 5.83, and with probability density function [15,40]


$$\frac{\alpha \gamma }{\alpha -\gamma }\left(e^{-\gamma t}-e^{-\alpha t}\right),$$

which is close to a Gamma distribution with the same mean and standard deviation. We input this Gamma as the serial interval in EpiEstim, implicitly assuming that the generation time is equivalent to the serial interval, and use a sliding window of 16 days (twice as long as the generation time, and chosen to marry with the choice of gap between knots in the GAM-based method). The comparison between EpiEstim and the estimate obtained with the methodology proposed here for the SEIR model is presented in the bottom panel of figures 2 and 6 (purple line and ribbon for EpiEstim versus red ones for our estimates). Although the results of the two methods show some differences, which is to be expected given that the approaches are substantially different (e.g. EpiEstim does not account for a day-of-the-week effect, which probably leads to the shorter time-scale oscillations compared with our method), the results show a similar general scale and trend with similar coverage of the 95% CrI. Note that we plot the EpiEstim estimate as the start date of the estimation window plus the assumed incubation period to align with the GAM estimator.

## 4. Discussion

This work brings together semi-parametric GAM with traditional structured compartmental epidemic models, an approach that has been developed in parallel in other recent studies in the literature [26]. The GAM can be used to calculate an instantaneous growth rate from time-series data and has an advantage that it can parametrically incorporate day-of-the-week effects to avoid unnecessary aggregation (e.g. to weekly numbers or rolling averages) to work around reporting effects. Layering on top of the GAM mechanistic assumptions about disease epidemiology, we have shown we can generate an estimate for the control reproduction number—the number of people infected per case in a wholly susceptible population, but with mixing patterns modified by a control policy or spontaneous response to an outbreak—and the effective reproduction number—the number of people infected per case given current mixing patterns and, importantly, partial susceptibility in the population.

This method for calculating time-varying control and effective reproduction numbers compares well with the alternative approach given by EpiEstim and is verified by simulation studies. Application to real-world data is promising. However, care homes are home to elderly people with strong staff contacts and it is not clear what impact age has on the mechanics of disease progression. Therefore, these estimates are mainly illustrative of the methodology, and further work is needed to compare them with the transmission estimates from community surveillance. The method shown here is applied to data about active outbreaks and case data, but it can be adapted to mortality data (see electronic supplementary material, section 2).

Limitations include not incorporating time-varying case fatality ratio δ and community immunity θ that in reality are known to have changed during the pandemic. However, all methods are sensitive to these changes and by parametrically including these effects one can be explicit in the assumptions for future interrogation. A second limitation is that single-type compartmental models assume homogeneous mixing in the population which means the method may be biased if the outbreak had core groups sustaining transmission. This is also true of other established tools such as EpiEstim.

At present, the uncertainty reflects only the inherent uncertainty from the GAM rather than uncertainty associated to the compartmental model parameter estimates. Furthermore, the SEIR model used assumes Markovian transitions between stages. This is a simplification but, as stated above, the intention is to get a representative metric for instantaneous transmission to aid decision makers’ thinking. Other model structures may be included at the cost of increased complexity in computation, uncertainty in output and subject to the inversion criteria stated in [26].

The method requires the specification of an initial condition I(0). The choice of I(0) affects the estimate of ρ towards the beginning of the time series, though its impact wanes on the timescale of a generation time. However, during this transient period, the estimate of ρ may need careful interpretation. The robustness of the results could be improved by incorporating uncertainty in I(0) as part of the inference scheme developed here or allowing the estimate of ρ to be presented only after a time period comparable with the generation time has elapsed (as is the case with EpiEstim).

We have derived a theoretical estimator of a dynamically changing population but shown results assuming the population is constant. Therefore these results also depend on the choice of the initial condition for the immune population R(0). The impact of this choice on our estimator would wane over time if we allow demographic change. However, given the pandemic context, it is reasonable to set R(0)=0 and let the immunity build up as more cases get infected. There needs to be some critical reflection on the derived estimator of $R_{C}$, though, given the weak capture of cases during the first wave (February–April 2020). We could infer this from calculation of excess mortality in the first wave [41], but this was not the focus of this paper. For simplicity and to show the method, we are accepting the data as an accurate reflection of the number of individuals infected in care homes over entire time series. However, we acknowledge that, due to low case ascertainment in the first wave, there may have been a larger depletion of susceptible population than we have accounted for here. Estimates of the effective reproduction number are not affected, but an underestimation of the immune population translates into an underestimation of the control reproduction number. Therefore, more careful considerations would be needed for quantitatively robust estimates, but we refrain from expanding on this issue here, given the main purpose of this work is to illustrate the methodology.

Case ascertainment may be an issue more generally. Transmission may not be identifiable if ascertainment rates were to vary over time independently of transmission changes. Moreover, the electronic supplementary material, section 3, shows via simulation that under-reporting of cases, even when constant over duration of reporting, can be an issue, as it appears to dilute the signal of transmission given it leads to smaller case numbers with larger relative intrinsic stochastic noise, and hence rates of changes in the data that are more uncertain. Work will continue to investigate the issues resulting from under-reporting, although again this is likely to be a problem also for the other methods available.

Diseases such as COVID-19 exhibit asymptomatic infection (cases who manifest no symptoms can still infect—or, if testing is available, cases might infect even prior to being detectable). In this study, we have assumed that all infections are detectable for simplicity in illustrating the methodology, but in the electronic supplementary material, Section 4, we relax this assumption to consider an infectious state prior to potential detection.

Application of EpiEstim to real-world datasets prior to the COVID-19 pandemic has focused on shorter time series. While we have not considered the length of the time series in detail, the GAM will require a certain amount of data to ascertain a valid fit and so caution may be needed in applications to shorter outbreaks than the ones analysed here.

The promising results from this method will be extended to consider simultaneous estimation of parameters across different datasets (i.e. calibrating to death data and case data, or to settings with nested populations, to infer subpopulation-specific reproduction numbers). Further work is also needed to explore full Bayesian approaches to the GAM inference to compare credible intervals and consider prediction from, and real-time application of, the models [26]. The splines derived have a fixed gap between knots, and adaptive grids may be worth exploring if over-/under-fitting can be controlled for. Here, we also assume structurally that the negative binomial scale parameter is constant. This may not be the case and more complex GAM forms may improve the results and the ensuing advice.

This method for estimating the time-varying reproduction number is effective and comparable to other methods. With appropriate handling the method is flexible to the type of epidemic impact measure provided (e.g. mortality or incidence), and it provides an alternative approach to smoothing out surveillance day-of-the-week effects to develop a measure of transmission that can enable action.

## Data Availability

Data and code to generate simulations and estimations and recreate plots in this paper can be accessed from [42]. Supplementary material is available online [43].
